# Apparatus for Administering the Letheon Vapour

**Published:** 1847-02

**Authors:** 


*Apparatus for Administering the Letheon Vapour.*—We promised to describe this apparatus in the present number. By reference, however, to the article ef H. I. Bigelow, M. D., in our last number, a general description will be found. They who wish to avail themselves of the method, will probably find it most convenient, for the present, to order an apparatus from Boston, where they are kept ready made. The expense is about $10. It is important that the Sulphuric Ether should be very pure.

